# Novel risk scoring system for predicting acute respiratory distress syndrome among hospitalized patients with coronavirus disease 2019 in Wuhan, China

**DOI:** 10.1186/s12879-020-05561-y

**Published:** 2020-12-17

**Authors:** Mengyuan Liang, Miao He, Jian Tang, Xinliang He, Zhijun Liu, Siwei Feng, Ping Chen, Hui Li, Yu’e Xue, Tao Bai, Yanling Ma, Jianchu Zhang

**Affiliations:** 1 Department of Respiratory and Critical Care Medicine, Union Hospital, Tongji Medical Collage, Huazhong University of Science and Technology, 1277 Jiefang Avenue, Wuhan, 430022 Hubei Province China; 2 Department of Neurology, Union Hospital, Tongji Medical Collage, Huazhong University of Science and Technology, 1277 Jiefang Avenue, Wuhan, 430022 Hubei Province China; 3 Department of Gastroenterology, Union Hospital, Tongji Medical Collage, Huazhong University of Science and Technology, 1277 Jiefang Avenue, Wuhan, 430022 Hubei Province China

**Keywords:** COVID-19, ARDS, Risk score

## Abstract

**Background:**

The mortality rate from acute respiratory distress syndrome (ARDS) is high among hospitalized patients with coronavirus disease 2019 (COVID-19). Hence, risk evaluation tools are required to immediately identify high-risk patients upon admission for early intervention.

**Methods:**

A cohort of 220 consecutive patients with COVID-19 were included in this study. To analyze the risk factors of ARDS, data obtained from approximately 70% of the participants were randomly selected and used as training dataset to establish a logistic regression model. Meanwhile, data obtained from the remaining 30% of the participants were used as test dataset to validate the effect of the model.

**Results:**

Lactate dehydrogenase, blood urea nitrogen, D-dimer, procalcitonin, and ferritin levels were included in the risk score system and were assigned a score of 25, 15, 34, 20, and 24, respectively. The cutoff value for the total score was > 35, with a sensitivity of 100.00% and specificity of 81.20%. The area under the receiver operating characteristic curve and the Hosmer–Lemeshow test were 0.967 (95% confidence interval [CI]: 0.925–0.989) and 0.437(*P* Value = 0.437). The model had excellent discrimination and calibration during internal validation.

**Conclusions:**

The novel risk score may be a valuable risk evaluation tool for screening patients with COVID-19 who are at high risk of ARDS.

**Supplementary Information:**

The online version contains supplementary material available at 10.1186/s12879-020-05561-y.

## Background

In late December 2019, an outbreak of pneumonia of unknown etiology first occurred inWuhan, China, and rapidly spread worldwide [1, 2]. Later studies confirmed that the disease was caused by a novel coronavirus referred to as severe acute respiratory syndrome coronavirus 2 (SARS-CoV- 2) [3, 4]. Then, in February 2020, this emerging infectious disease was officially named as coronavirus disease 2019 (COVID-19) by the World Health Organization (WHO) [5, 6]. Although most patients diagnosed with COVID-19 experience mild symptoms, 19% could develop severe or fatal symptoms and present with intractable conditions, particularly ARDS [7]. With respect to its definition, the pathogenesis of ARDS involves rapidly progressing respiratory failure from non-cardiogenic pulmonary edema, which may require mechanical ventilation due to severe hypoxia and difficulty in breathing [8]. Currently, the Berlin definition for ARDS is utilized, and it recommends the use of three categories to differentiate severity based on partial pressure of oxygen (PaO2)/initial fraction of inspired oxygen (FiO2) [9]. According to a recently published study, the mortality rate of patients with COVID-19 who present with ARDS is > 70% [10]. Moreover, the current guidelines for the treatment of ARDS focus on lung-protective ventilation and fluid conservative management, and early interventions were found to have a better therapeutic effect [11]. Thus, to facilitate the early identification of high-risk patients and prevent the development of or reduce the severity of ARDS, the predictors of this condition must be determined. Recent studies have identified several predictors for the unfavorable outcomes of COVID-19 [12–14]. However, only few predictors of the onset of ARDS were determined. Thus, the key indicators for the risk of ARDS should be immediately identified upon admission. Moreover, risk evaluation models that use a combination of risk factors are likely to increase the power of prediction. Some risk scoring systems have been developed for several clinical conditions, including coronary heart disease [15], heart failure [16], and stroke [17], and these systems were found to have great practical value. Thus, a systemic evaluation tool involving risk scores, which can be practical for clinicians, is urgently needed. In this study, we obtained data from 220 patients with confirmed COVID-19 who died in or were discharged from the isolation ward of the Department of Respiratory and Critical Care Medicine at Wuhan Union Hospital between January 10, 2020, and March 5, 2020. The current study aimed to explore the risk factors associated with ARDS in patients with COVID-19 and develop a risk evaluation system for predicting ARDS.

## Methods

### Study design and data collection

This was a retrospective, observational cohort study performed at the isolation ward of the Department of Respiratory and Critical Care Medicine, Wuhan Union Hospital (Huazhong University of Science and Technology, Wuhan, China). We included all adult patients who were diagnosed with COVID-19, according to the WHO interim guidance, and who were discharged or died between January 10, 2020, and March 5, 2020. Since Wuhan Union Hospital has been a designated hospital for treating patients with COVID-19 since January 10, 2020, the population constituted a representative sample of all patients with COVID-19 seeking treatment. This study was approved by the research ethics commission of Wuhan Union Hospital (KY-2020-0040), and the need for informed consent was waived. Demographic, clinical, laboratory, and imaging data were extracted from the electronic medical record system of Wuhan Union Hospital through a standardized data collection form. All data were checked by two physicians (YM and JT), and a third researcher (MH) reviewed and made corrections to any differences in data.

### Case definition

COVID-19 was confirmed based on the examination of respiratory specimens using real-time reverse transcription polymerase chain reaction (RT-PCR). The examination was performed at the clinical laboratory of Wuhan Union Hospital, which is a qualified institution for nucleic acid testing. Patients who were discharged met all of the following criteria: 1) absence of fever for at least 3 days, 2) notable improvement of findings on chest computed tomography (CT) scan, 3) remission of respiratory symptoms, and 4) two continuous negative results of SARS-CoV-2 RNA using throat swab samples collected at least 24 h apart.

Fever was defined as an axillary temperature of at least 37.3 °C. ARDS was diagnosed according to the Berlin Definition [9]. In brief, patients who experienced acute respiratory failure not fully explained by cardiac failure or fluid overload, with PaO2/FiO2 ≤ 300 mmHg and positive end expiratory pressure or continuous positive airway pressure ≥ 5 cm H2O, and who present with bilateral opacities on chest radiography not fully explained by effusions, lobar or lung collapse, or nodules are diagnosed with ARDS [8, 9].

### Clinical examinations and treatments

Routine blood examinations, including complete blood count, liver function, blood lipids, fasting blood glucose, kidney function, uric acid, lactate dehydrogenase, creatine kinase, and assessment of myocardial enzymes, coagulation profile, serum C-reactive protein (CRP) level, erythrocyte sedimentation rate (ESR), serum procalcitonin (PCT) level, and ferritin level, were performed upon patient admission, and re-examination was conducted at least once every 3 days during hospitalization. All patients who were admitted underwent chest CT scans.

Patients admitted to the isolation ward received standard treatment according to the Chinese management guidelines for COVID-19 (version 6.0) [18]. In brief, the antiviral treatment included interferon alpha inhalation (50 μg twice daily), lopinavir, and ritonavir (400 and 100 mg twice daily, respectively), and arbidol (200 mg twice daily). Treatment with corticosteroid (40–80 mg/day) and gamma globulin (15–20 g/day) was initiated if patients presented with a resting respiratory rate > 30 per min, oxygen saturation < 93% but without the need for supplemental oxygen, or chest radiography showing > 50% progression within 48 h. Oral and intravenous antibiotics were administered if there was a high risk of concomitant bacterial infection.

### Statistical methods

Data obtained from approximately 70% of the participants (*n* = 154) were randomly selected and used as training dataset to establish a logistic regression model. Meanwhile, data obtained from the remaining 30% of the patients (*n* = 66) were used as test dataset to validate the effect of the model. Using the training dataset, continuous and categorical variables were presented as median with interquartile range (IQR) and total number (n) with percentage (%), respectively. The Mann–Whitney U test, χ2 test, and Fisher’s exact test were used accordingly to compare the differences between patients with and without ARDS. Univariate and multivariate logistic regression models were used to test the association between the risk factors and onset of ARDS. A receiver operating characteristic (ROC) curve was established to depict the predictive ability of the variables. The Youden index, which was calculated as the sum of the sensitivity and specificity minus 1, was used to determine the optimal cutoff value. The area under the curve (AUC) was calculated with the ROC curves to determine the differentiating abilities of the corresponding risk factors. Variables with significant differences between patients with and without ARDS were included in the multivariate logistic regression analysis, and a stepwise selection method was used to identify the variables included in the predictive model. The assigned risk score for the corresponding variables was determined by multiplying the β coefficients of significant variables by 10 and rounding off the value to the nearest integers, and the total risk score was calculated as the sum of those of the individual risk factors. In the verification dataset, the model was evaluated using the Hosmer–Lemeshow test and ROC curve. All data analyses were performed using SAS version 9.4 (SAS Institute Inc., Cary, NC, the USA). Two-tailed *P* values < 0.05 for all tests were considered statistically significant. *P* values < 0.1 were used as the selection criteria for variables in the model.

## Results

Of the 154 COVID-19 patients whose data were used to establish the model, 37 (24.03%)developed ARDS during hospitalization. Data about the characteristics of the study population collected upon admission and grouped according to the diagnosis of ARDS are presented in Table 1.

**Table 1:** Characteristics of the study population

	Total, (***n*** = 154)	ARDS, (***n*** = 37)	Non-ARDS, (***n*** = 117)	***P*** value
**Demographics and clinical characteristics**				
**Age (years)**	57.5 (45–70)	69 (62–74)	54 (41–65)	< 0.001***
**Gender, n (%)**				
**Male**	83 (53.90%)	26 (70.27%)	57 (48.72%)	0.022*
**Female**	71 (46.10%)	11 (29.73%)	60 (51.28%)	
**Smoking habits, n (%)**	13 (13.64%)	9 (24.32%)	12 (10.26%)	0.030*
**Weight (kg)**	65.0 (55.0–75.0)	63.5 (55.0–73.0)	65.0 (55.5–82.0)	0.511
**Fever, n (%)**	138 (89.61%)	34 (91.89%)	104 (88.89%)	0.832
**Cough, n (%)**	102 (66.23%)	21 (56.76%)	81 (69.23%)	0.162
**Diarrhea, n (%)**	17 (11.04%)	4 (10.81%)	13 (11.11%)	0.959
**Fatigue, n (%)**	109 (70.78%)	28 (75.68%)	81 (69.23%)	0.452
**Dyspnea, n (%)**	62 (40.26%)	14 (37.84%)	48 (41.03%)	0.730
**Time from symptoms onset to hospitalization, d**	10 (6–13)	8 (6–11)	10 (6–15)	0.059
**Comorbidity, n (%)**	68 (44.16%)	27 (72.97%)	41 (35.04%)	< 0.001***
**Hypertension, n (%)**	43 (27.92%)	16 (43.24%)	27 (23.08%)	0.017*
**Diabetes, n (%)**	28 (18.18%)	13 (35.14%)	15 (12.82%)	0.002**
**CHD, n (%)**	14 (9.09%)	6 (16.22%)	8 (6.84%)	0.161
**Malignancy, n (%)**	6 (3.09%)	4 (10.81%)	2 (1.71%)	0.045*
**Laboratory findings**				
**White blood cell (×10**^**9**^**/L)**	4.845 (3.71–6.52)	6.50 (4.79–10.26)	4.64 (3.50–6.00)	< 0.001***
**Lymphocyte (×10**^**9**^**/L)**	0.975 (0.67–1.38)	0.53 (0.40–1.07)	1.03 (0.78–1.52)	< 0.001***
**Hemoglobin(g/L)**	126 (115–137)	129 (119–144)	126 (114–137)	0.136
**ALT (U/L)**	33.5 (19–54)	44 (31–65)	28 (18–48)	0.002**
**AST (U/L)**	34 (25–47)	48 (37–79)	30 (23–41)	< 0.001***
**LDH (U/L)**	262 (198–353)	415 (307–594)	233.5 (185–291.5)	< 0.001***
**Albumin (g/L)**	32.35 (28.9–35.80)	29.4 (26.5–33.0)	33.7 (29.9–36.5)	< 0.001***
**Creatinine (μmol/L)**	69.4 (58.4–83.6)	74.5 (65.5–103.6)	66.9 (56.3–78.7)	0.004**
**Blood urea nitrogen (mmol/L)**	4.25 (3.30–5.91)	5.91 (4.50–10.90)	4.00 (3.13–4.93)	< 0.001***
**CK-MB (ng/mL)**	0.6 (0.4–1.1)	1.05 (0.8–2.4)	0.5 (0.3–0.9)	< 0.001***
**Troponin I (ng/L)**	5.0 (2.3–11.9)	14.15 (8.0–39.6)	3.65 (1.9–8.6)	< 0.001***
**Prothrombin time (s)**	13.4 (12.8–14.2)	13.9 (13.1–14.7)	13.3 (12.75–14.1)	0.033*
**D-Dimer (μg/ml)**	0.50 (0.31–1.58)	1.88 (0.62–8.00)	0.465 (0.24–0.945)	< 0.001***
**C-Reactive protein (mg/L)**	23.395 (4.675–59.565)	61.69 (41.63–104.38)	9.58 (2.71–37.20)	< 0.001***
**ESR (mm/h)**	48.5 (29–73)	64.0 (38.5–73.5)	45.5 (25–70.5)	0.046*
**Procalcitonin (ng/ml)**	0.09 (0.05–0.21)	0.26 (0.14–0.63)	0.07 (0.04–0.12)	< 0.001***
**Ferritin (ng/ml)**	509.32 (226.86–937.73)	1305.325 (664.355–2000.00)	416.72 (190.165–692.415)	< 0.001***
**Imaging features**				
**Consolidation, n (%)**	48 (31.17%)	9 (24.32%)	39 (33.33%)	0.302
**Bilateral infiltration, n (%)**	144 (93.51%)	34 (91.89%)	110 (94.02%)	0.941
**Outcome**				
**Death**	16 (10.39%)	15 (40.54%)	1 (0.85%)	< 0.001***

Mann-Whitney U tests were used to detect differences of continuous variables between ARDS and non-ARDS group. Chi-squared tests or Fisher’s exact tests were used to detect difference of categorical variables between ARDS and non-ARDS group

*p-value< 0.05

**p-value< 0.01

***p-value< 0.001

The median age of the patients was 57.5 (range: 21–96, IQR: 45–75) years. The median age of the ARDS group was significantly higher than that of the non-ARDS group (69 [IQR: 62–74] vs 54 years [IQR 41–65] years). Approximately 53.90% of the patients were men. The male patients accounted for a larger proportion in the ARDS group (70.27%) than in the non-ARDS group (48.72%). About 13.64% of the patients were smokers, and the proportion of smokers was larger in the ARDS group (24.32%) than in the non-ARDS group (10.26%). The patients in the ARDS and non-ARDS groups did not differ significantly in terms of weight and clinical features upon admission. With respect to clinical features, fever was the most common symptom upon admission that was observed in 89.61% of the total population. The median time from symptoms onset to hospitalization in the ARDS group and non-ARDS group was 8 days and 10 days, respectively and there was no statistical difference. Moreover, patients with comorbidities were more likely to develop ARDS than patients without comorbidities (72.97% vs. 35.04%). The comorbidities included hypertension (43.24% vs 23.08%), diabetes (35.14% vs. 12.82%), coronary heart disease (CHD, 16.22% vs. 6.84%), and malignancies (10.81% vs. 1.71%). However, the difference was not significant in terms of the prevalence of CHD.

In terms of the results of the laboratory examinations performed upon admission, COVID-19 patients with ARDS had significantly higher levels of white blood cell (WBC: 6.50 × 109 /L, IQR: 4.79–10.26 vs. 4.64 × 109 /L, IQR: 3.50–6.00), alanine transaminase (ALT: 44 U/L, IQR: 31–65 vs. 28 U/L, IQR: 18–48), aspartate aminotransferase (AST: 48 U/L, IQR: 37–79 vs. 30 U/L, IQR: 23–41), lactate dehydrogenase (LDH: 415 U/L, IQR: 307–594 vs. 233.5 U/L, IQR: 185–291.5), creatinine (74.5 μmol/L, IQR: 65.5–103.6 vs. 66.9 μmol/L, IQR: 56.3–78.7), blood urea nitrogen (BUN: 5.91 mmol/L, IQR: 4.50–10.90 vs. 4.00 mmol/L, IQR: 3.13–4.93), creatine kinase-MB (CK-MB: 1.05 ng/mL, IQR: 0.8–2.4 vs. 0.5 ng/mL, IQR: 0.3–0.9), highly sensitive troponin I (14.15 ng/L, IQR: 8.0–39.6 vs. 3.65 ng/L, IQR: 1.9–8.6), prothrombin time (PT: 13.9 s, IQR: 13.1–14.7 vs. 13.3 s, IQR: 12.75–14.1), D-dimer (1.88 μg/mL, IQR: 0.62–8.00 vs. 0.465 μg/mL, IQR: 0.24–0.945), CRP (61.69 mg/L, IQR: 41.63–104.38 vs. 9.58 mg/L, IQR: 2.71–37.20), ESR (64.0 mm/h, IQR: 38.5–73.5, vs. 9.58 mm/h, IQR: 25–70.5), PCT (0.26 ng/mL, IQR: 0.14–0.63 vs. 0.07 ng/mL, I-QR: 0.04–0.12), and ferritin (1305.325 ng/mL, IQR: 664.355–2000.00 vs. 416.72 ng/mL, IQR: 190.165–692.415) than COVID-19 patients without ARDS. Moreover, COVID-19 patients with ARDS had lower levels of lymphocytes (0.53 × 109 /L, IQR: 0.40–1.07 vs 1.03 × 109 /L, IQR: 0.78–1.52) and serum albumin (ALB: 29.4 g/L, IQR: 26.5–33.0 vs 33.7 g/L, IQR: 29.9–36.5) than COVID-19 patients without ARDS. However, there was no significant difference between the ARDS and non-ARDS groups in terms of consolidation and bilateral pulmonary infiltration on chest CT scans.

Regarding other important indicators such as PaO2 and PaO2/FiO2 at admission, We only recorded the above indicators for 29 patients, of which 28 patients were ARDS patients and 1 was non-ARDS patient. The median PaO2 of ARDS patients is 64 mmHg, PaO2/FiO2 is 101 mmHg.the Non-ARDS patient is 81 mmHg and 245 mmHg, respectively. Although the ARDS group has worse indicators, the data is insufficient, resulting in data differences between the two groups that are not comparable.

The mortality in the ARDS group was 40.54%, while the mortality in the non-ARDS group was much lower than that of the ARDS group, which was 0.85%. This indicates that the ARDS group has a higher risk of death, and also shows the importance of the risk assessment model, which provides a basis for early detection and early treatment.

Table 2 shows the predictive efficiency of continuous variables that were differentially distributed between the ARDS and non-ARDS groups. The cutoff criteria were selected with the best Youden indices, and the associated sensitivities, specificities, positive likelihood ratios (+LR), and negative likelihood ratios (−LR) were also presented. The AUC values were determined using the ROC curves, as shown in Figure S1. Among the continuous risk factors, ferritin level had the best differentiating ability, with an AUC of 0.872, and the associated cutoff value was > 950 ng/mL.

**Table 2:** Predictive efficiency of continuous risk factors for developing ARDS

Variables	Youden Index	Cut-off criterion	Sensitivity	Specificity	+LR	-LR	AUC (95%CI)
Age (years)	0.419	≥ 65	67.57%	74.36%	2.64	0.44	0.725 (0.647–0.793)
White blood cell (×10^9^/L)	0.363	> 6.10	59.46%	76.92%	2.58	0.53	0.714 (0.635–0.783)
Lymphocyte (× 10^9^/L)	0.496	≤0.71	67.57%	82.05%	3.76	0.40	0.766 (0.691–0.830)
ALT (U/L)	0.332	≥ 30	81.08%	52.14%	1.69	0.36	0.676 (0.596–0.749)
AST (U/L)	0.423	≥ 37	75.68%	66.67%	2.27	0.36	0.772 (0.697–0.835)
LDH (U/L)	0.597	≥ 295.0	83.78%	75.86%	3.47	0.21	0.860 (0.795–0.911)
Albumin (g/L)	0.304	< 28.5	43.24%	87.18%	3.37	0.65	0.685 (0.605–0.757)
Creatinine (μ mol/L)	0.340	≥ 88.0	45.95%	88.03%	3.84	0.61	0.659 (0.579–0.734)
BUN (mmol/L)	0.479	> 5.12	67.57%	80.34%	3.44	0.40	0.763 (0.688–0.828)
CK-MB (ng/mL)	0.498	> 0.70	77.78%	72.00%	2.78	0.31	0.803 (0.726–0.866)
Troponin I (ng/L)	0.507	> 4.70	88.89%	61.76%	2.32	0.18	0.821 (0.746–0.881)
Prothrombin time (s)	0.209	> 14.20	37.84%	83.04%	2.23	0.75	0.618 (0.535–0.697)
D-Dimer (μ g/ml)	0.307	≥ 5.00	32.43%	98.21%	18.16	0.69	0.691 (0.611–0.764)
C-Reactive protein (mg/L)	0.639	≥ 38.00	86.49%	77.39%	3.83	0.17	0.852 (0.785–0.904)
ESR (mm/h)	0.294	≥60.0	63.89%	65.52%	1.85	0.55	0.611 (0.529–0.689)
Procalcitonin (ng/ml)	0.581	> 0.11	83.78%	74.31%	3.26	0.22	0.854 (0.787–0.907)
Ferritin (ng/ml)	0.602	≥ 950.00	69.44%	90.74%	7.50	0.34	0.872 (0.806–0.921)

*p-value< 0.05

**p-value< 0.01

***p-value< 0.001

**Fig. 1:**
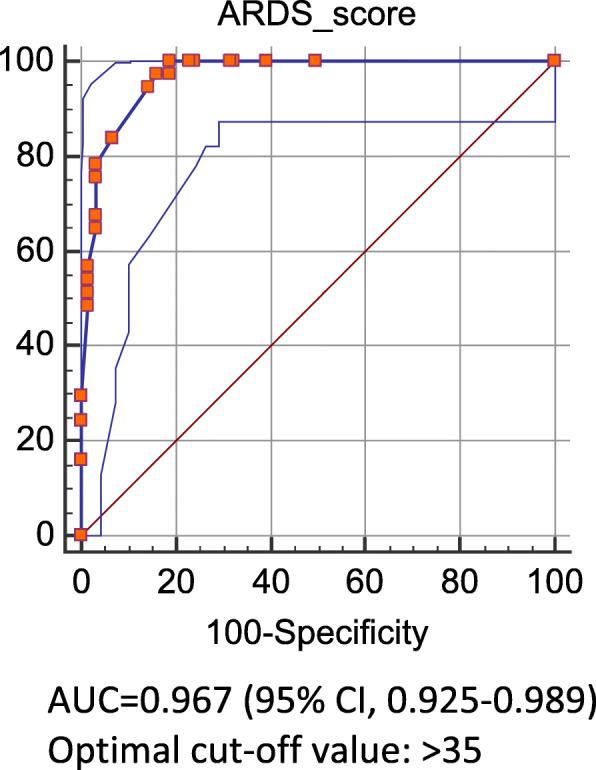
ROC curve of the risk score for predicting ARDS

The results for the association between differentially distributed risk factors and the risk of ARDS are shown in Table 3. The unadjusted models were first used to analyze the risk of individual variables. Then, the multivariate logistic regression model was fitted with a stepwise selection method, and LDH, BUN, D-dimer, PCT, and ferritin levels were selected and used in the combined prediction model. Specifically, COVID-19 patients with LDH values ≥295 U/L were assigned a score of 25; those with BUN values > 5.12 mmol/L, a score of 15; D-dimer values ≥5.00 μg/mL, a score of 34; PCT values > 0.11 ng/mL, a score of 20; and serum ferritin values ≥950 ng/mL, a score of 24. The total risk scores were calculated as the sum of the scores of the individual risk factors. The ROC curves of the total risk score for predicting ARDS are depicted in Fig. 1, and the associated sensitivities, specificities, +LRs, and –LRs of each cutoff point are presented in Table S1. In the current study cohort, the optimal cutoff value of the ARDS risk score was > 35, with a sensitivity of 100% and a specificity of 81.20%. The AUC of the ARDS score based on the ROC curve was 0.967 (95% CI: 0.925–0.989).

**Table 3:** Risk factors for developing ARDS among hospitalized COVID-19 patients

Variables	Univariable OR (95%CI)	*P* value	Multivariable OR (95%CI)	*P* value	β coefficients	Assigned risk score
Age (years)						
< 65	1(reference)	…	…	…	…	…
≥ 65	6.041 (2.704–13.496)	< 0.001***	…	…	…	…
Male (vs female)	2.488 (1.126–5.497)	0.024*	…	…	…	…
Smoking (vs non-smoker)	2.813(1.077–7.342)	0.035*	…	…	…	…
Comorbidity (vs not present)	5.005 (2.207–11.351)	< 0.001***	…	…	…	…
Hypertension (vs not present)	2.540 (1.165–5.539)	0.019*	…	…	…	…
Diabetes (vs not present)	3.683 (1.550–8.752)	0.003**	…	…	…	…
CHD (vs not present)	2.637 (0.851–8.173)	0.093	…	…	…	…
Malignancy (vs not present)	6.966 (1.222–39.717)	0.029*	…	…	…	…
White blood cell (×10^9^/L)						
≤ 6.10	1(reference)	…	…	…	…	…
> 6.10	4.889 (2.231–10.715)	< 0.001***	…	…	…	…
Lymphocyte (×10^9^/L)						
> 0.71	1(reference)	…	…	…	…	…
≤ 0.71	9.523 (4.133–21.941)	< 0.001***	…	…	…	…
ALT (U/L)						
< 30	1(reference)	…	…	…	…	…
≥ 30	4.667 (1.899–11.469)	< 0.001***	…	…	…	…
AST (U/L)						
< 37	1(reference)	…	…	…	…	…
≥ 37	6.221 (2.676–14.462)	< 0.001***	…	…	…	…
LDH (U/L)						
< 295.0	1(reference)	…	1(reference)	…	…	…
≥ 295.0	20.342 (7.235–57.192)	< 0.001***	11.867 (2.569–54.815)	0.002**	2.474	25
Albumin (g/L)						
≥ 28.5	1(reference)	…	…	…	…	…
< 28.5	5.181 (2.222–12.081)	< 0.001***	…	…	…	…
Creatinine (μ mol/L)						
< 88.0	1(reference)	…	…	…	…	…
≥ 88.0	6.254 (6.254–14.693)	< 0.001***	…	…	…	…
BUN (mmol/L)						
≤ 5.12	1(reference)	…	1(reference)	…	…	…
> 5.12	8.514 (3.729–19.439)	< 0.001***	4.706 (1.160–19.098)	0.030*	1.549	15
CK-MB (ng/mL)						
≤ 0.70	1(reference)	…	…	…	…	…
> 0.70	9.889 (4.173–23.434)	< 0.001***	…	…	…	…
Troponin I (ng/L)						
≤ 4.70	1(reference)	…	…	…	…	…
> 4.70	12.800 (4.625–35.421)	< 0.001***	…	…	…	…
Prothrombin time (s)						
≤ 14.20	1(reference)	…	…	…	…	…
> 14.20	3.140 (1.374–1.374)	0.007**	…	…	…	…
D-Dimer (μg/ml)						
< 5.00	1(reference)	…	1(reference)	…	…	…
≥ 5.00	35.000 (7.445–164.531)	< 0.001***	30.001 (2.282–394.374)	0.010*	3.401	34
C-Reactive protein (mg/L)						
< 38.00	1(reference)	…	…	…	…	…
≥ 38.00	22.398 (7.930–63.267)	< 0.001***	…	…	…	…
ESR (mm/h)						
< 60.0	1(reference)	…	…	…	…	…
≥ 60.0	3.162 (1.470–6.806)	0.003**	…	…	…	…
Procalcitonin (ng/ml)						
≤ 0.11	1(reference)	…	1(reference)	…	…	…
> 0.11	16.422 (6.214–43.402)	< 0.001***	7.295 (1.772–30.042)	0.006**	1.987	20
Ferritin (ng/ml)						
< 950.00	1(reference)	…	1(reference)	…	…	…
≥ 950.00	22.292 (8.661–57.377)	< 0.001***	11.227 (2.904–43.400)	0.001**	2.4184	24

*p-value< 0.05

**p-value< 0.01

***p-value< 0.001

In this study, data obtained from the remaining 30% of the patients were used as validation dataset, In total, 17 of 66 patients had ARDS. The sensitivity of the model was 71.43% and specificity was 78.85%. The AUC of the test sample model was 0.819 (95% CI: 0.680–0.959). The *P* value for the Hosmer–Lemeshow test was 0.312, indicating that the model had a good fit (Table 4).

**Table 4:** Evaluation of ARDS risk assessment model

Predicating model	Berlin definition of ARDS		Total
	ARDS	Non-ARDS	
**High risk**	10	11	21
**Low risk**	4	41	45
**Total**	14	52	66

## Discussion

This is a retrospective study of patients diagnosed with COVID-19 and admitted to Wuhan Union Hospital. By comparing patients with and without ARDS, a panel of risk factors were identified. The univariate logistic regression model was used to assess the risk of individual factors, and the multivariate logistic regression model was utilized to identify the factors for the risk prediction model, which include LDH, BUN, D-dimer, PCT, and serum ferritin levels. Moreover, by assigning a risk score for the significant factors and calculating the total risk score, COVID-19 patients with a high risk of ARDS during hospitalization could be identified and this risk evaluation system had a good predictive efficiency.

Among the variables included in the prediction model, the D-dimer level was assigned with the largest risk score at 34. Although the underlying mechanism of COVID-19 is still unknown, patients with this condition have an increased risk of thrombosis (preliminary data not shown). Bronchoscopy showed red jelly-like sputum, and biopsy of the lung tissues revealed disseminated hemorrhage in the pulmonary alveoli and clot formation within the microvessels [19, 20]. This result emphasizes the role of blood clotting dysfunction, as indicated by elevated D-dimer levels. Intra-alveoli hemorrhage and intravascular thrombosis are bound to reduce gas exchange function, and this leads to severe hypoxia, which is considered a key manifestation of ARDS [8].

In previous studies, an elevated ferritin level was considered a risk factor for the severity of different types of infection [21–23]. Although several studies have compared the difference in risk factors between patients with favorable and unfavorable outcomes, sufficient attention has not been paid to ferritin levels [10, 13, 24]. One recent study reported that the non-survivors of COVID-19 had higher serum ferritin levels than survivors [25]. By contrast, the formation of toxic hydroxyl radicals from superoxide anions and hydrogen peroxide requires free iron, the storage of which correlates with that of ferritin. In contrast, proinflammatory cytokines, including interleukin-1β (IL-1β), interleukine-6 (IL-6), and tumor necrosis factor-α, can directly increase the synthesis of ferritin [26].

ARDS is characterized by acute, diffuse, inflammatory lung injury; and inflammatory markers, including CRP, ESR, PCT, and serum ferritin, may worsen the clinical symptoms [27, 28]. In addition to serum ferritin, PCT was included in the prediction model. Although ferritin is considered a marker of tissue inflammation, PCT is more commonly considered as an indicator of bacterial infection [29, 30]. This result indicates that concomitant bacterial infection may play an important role in the progression of ARDS among patients with COVID-19, and this finding is in accordance with a previous hypothesis on the pathogenesis of the disease [31–33]. Thus, for high-risk patients with elevated PCT levels, treatment with antibiotics may be effective in preventing ARDS. Although we have not obtained precise etiological evidence, we found that the Procalcitonin in the ARDS group was higher and exceeded the normal range, accompanied by an increase in White blood cell count, C-Reactive protein, ESR, Ferritin and a decrease in lymphocyte count. This also suggests that it seems that patients in the ARDS group have worse immunity and a higher risk of bacterial/fungal co-infection.

Since SARS-CoV2 attack pulmonary epithelial cells and there is a risk of bacterial infection, the release of intracellular LDH, which is considered a general index of cell injury, is bound to increase [34, 35]. Moreover, recent studies have revealed that lactate may suppress the function of immune cells, and LDH can be an indicator of immunosuppression [36, 37]. Thus, special attention must be paid to this finding, as the current research and previous reports [10, 25] have shown that a decreased number of lymphocytes can be associated with unfavorable outcomes among patients with COVID-19. This result indicated that immune suppression had an important role in disease prognosis. However, in our model, the addition of lymphocytes does not improve the predictive efficiency of the model, indicating that the effect of lymphocytes on ARDS may be explained by other variables, including LDH.

Moreover, the importance of BUN in predicting ARDS has not received special attention. As severe infection and tissue damage can increase the rate of protein degradation [38], patients with ARDS had elevated BUN levels. Moreover, the cutoff value for BUN levels in predicting ARDS is > 5.12 mmol/L, which is still within the normal range. BUN levels higher than the normal range can indicate > 50% loss of renal function, and this may emphasize the preservation of renal function in patients with COVID-19, which plays a decisive role in fluid control for treatment of patients with ARDS [27]. With respect to the specific scores assigned to the individual risk factors, these variables may divided into groups with high scores, including those of LDH, D-dimer, and ferritin levels, with scores of 25, 34, and 24, respectively, and groups with low scores, including those of BUN and PCT, with scores of 15 and 20, respectively. As the optimal cutoff value is > 35, a positive result for any single factor is not sufficient to identify high-risk patients, and any combination of the three factors is sufficient. However, when evaluation is based on two variables, one should be included in the high-score group.

This study had several strengths. First, Wuhan Union Hospital is one of the first designated institutions for treating patients with COVID-19 in Wuhan. Thus, the participants constituted a representative sample of hospitalized patients. Moreover, this study had one of the largest populations with definite outcomes during hospitalization, thereby providing a strong evidence for depicting the risk factors of ARDS among patients with COVID-19. By contrast, after a systemic selection of possible risk factors, a panel of indices routinely tested in clinical settings were selected, and a risk evaluation score was designed with a relatively good predictive efficacy for practical use. However, this study also had several limitations. First, the interpretation of the results may be limited by the relatively small sample size. Second, studies involving external verification must be conducted to validate the efficacy of our model in predicting ARDS. Finally, Due to the heavy clinical work during the epidemic, some important indicators such as PaO2 and PaO2/FiO2 have not been recorded in time, so these indicators cannot be included in our research.

## Conclusions

Several variables were found to be differentially distributed between COVID-19 patients with and without ARDS. A rough stepwise selection method and a panel of risk factors, including LDH, BUN, D-dimer, PCT, and ferritin levels, were included in the prediction model, and a risk evaluation scoring system was established, with an optimal cutoff value > 35 and AUC of 0.967 (95% CI: 0.925–0.989). Moreover, the model had excellent discrimination and calibration during the internal validation, which is considered practical for clinicians. The novel risk scoring system may be a valuable tool for screening COVID-19 patients with a high risk of ARDS.

## Supplementary Information

**Additional file 1:** **Table S1**. Predictive efficiency of the ARDS risk score among hospitalized COVID-19 patients.

**Additional file 2:** **Figure S1.** ROC curves of individual risk factors for predicting ARDS.

## Data Availability

All Data and material collected during this study are available from the corresponding author upon reasonable request. Wuhan Union Hospital was as one of the first hospital received patients with COVID-19 in Wuhan, so the data of some patients in this study was likely to be used in other studies.

## Abbreviations

COVID-19: Coronavirus disease 2019
ARDS: Acute respiratory distress syndrome
ROC curve: Receiver operating characteristic curve
AUC: Area under the curve
LDH: Lactate dehydrogenase
BUN: Blood urea nitrogen
PCT: Procalcitonin
SARS-CoV-2: Severe acute respiratory syndrome coronavirus 2
RT-PCR: Reverse transcription-polymerase chain reaction; C-reactive protein
CRP: Erythrocyte sedimentation rate
ESR: PaO2 Partial pressure of atrial oxygen
FiO2: Fraction of inspired oxygen
PEEP: Positive end-expiratory pressure
CPAP: Continuous positive airway pressure
IQR: Interquartile range
CI: Confidence interval
OR: Odds ratio
