# Supplementary material for: Novel risk scoring system for predicting acute respiratory distress syndrome among hospitalized patients with coronavirus disease 2019 in Wuhan, China
Source: BMC Infect Dis. 2020 Dec 17;20:960. doi: 10.1186/s12879-020-05561-y (PMC7744733; doi:10.1186/s12879-020-05561-y)
Supplement: Supplementary file 1 — Additional file 1: Table S1. Predictive efficiency of the ARDS risk score among hospitalized COVID-19 patients. [file 12879_2020_5561_MOESM1_ESM.docx]

**Table S1. Predictive efficiency of the ARDS risk score among hospitalized COVID-19 patients**

| Score criterion | Sensitivity | Specificity | +LR | -LR |
| --- | --- | --- | --- | --- |
| ≥0 | 100.00% | 0.00% | 1.00 |  |
| >0 | 100.00% | 50.43% | 2.02 | 0.00 |
| >15 | 100.00% | 60.68% | 2.54 | 0.00 |
| >20 | 100.00% | 67.52% | 3.08 | 0.00 |
| >24 | 100.00% | 68.38% | 3.16 | 0.00 |
| >25 | 100.00% | 76.07% | 4.18 | 0.00 |
| >34 | 100.00% | 76.92% | 4.33 | 0.00 |
| >35 | 100.00% | 81.20% | 5.32 | 0.00 |
| >39 | 97.30% | 81.20% | 5.17 | 0.033 |
| >40 | 97.30% | 83.76% | 5.99 | 0.032 |
| >44 | 94.59% | 85.47% | 6.51 | 0.063 |
| >45 | 83.78% | 93.16% | 12.25 | 0.17 |
| >49 | 78.38% | 96.58% | 22.93 | 0.22 |
| >59 | 75.68% | 96.58% | 22.14 | 0.25 |
| >60 | 67.57% | 96.58% | 19.76 | 0.34 |
| >64 | 64.86% | 96.58% | 18.97 | 0.36 |
| >69 | 56.76% | 98.29% | 33.20 | 0.44 |
| >74 | 54.05% | 98.29% | 31.62 | 0.47 |
| >79 | 51.35% | 98.29% | 30.04 | 0.49 |
| >83 | 48.65% | 98.29% | 28.46 | 0.52 |
| >84 | 29.73% | 100.00% |  | 0.70 |
| >93 | 24.32% | 100.00% |  | 0.76 |
| >94 | 16.22% | 100.00% |  | 0.84 |
| >118 | 0.00% | 100.00% |  | 1.00 |
