# Supplementary material for: Novel risk scoring system for predicting acute respiratory distress syndrome among hospitalized patients with coronavirus disease 2019 in Wuhan, China
Source: BMC Infect Dis. 2020 Dec 17;20:960. doi: 10.1186/s12879-020-05561-y (PMC7744733; doi:10.1186/s12879-020-05561-y)
Supplement: Supplementary file 2 [file 12879_2020_5561_MOESM2_ESM.docx]

Figure S1

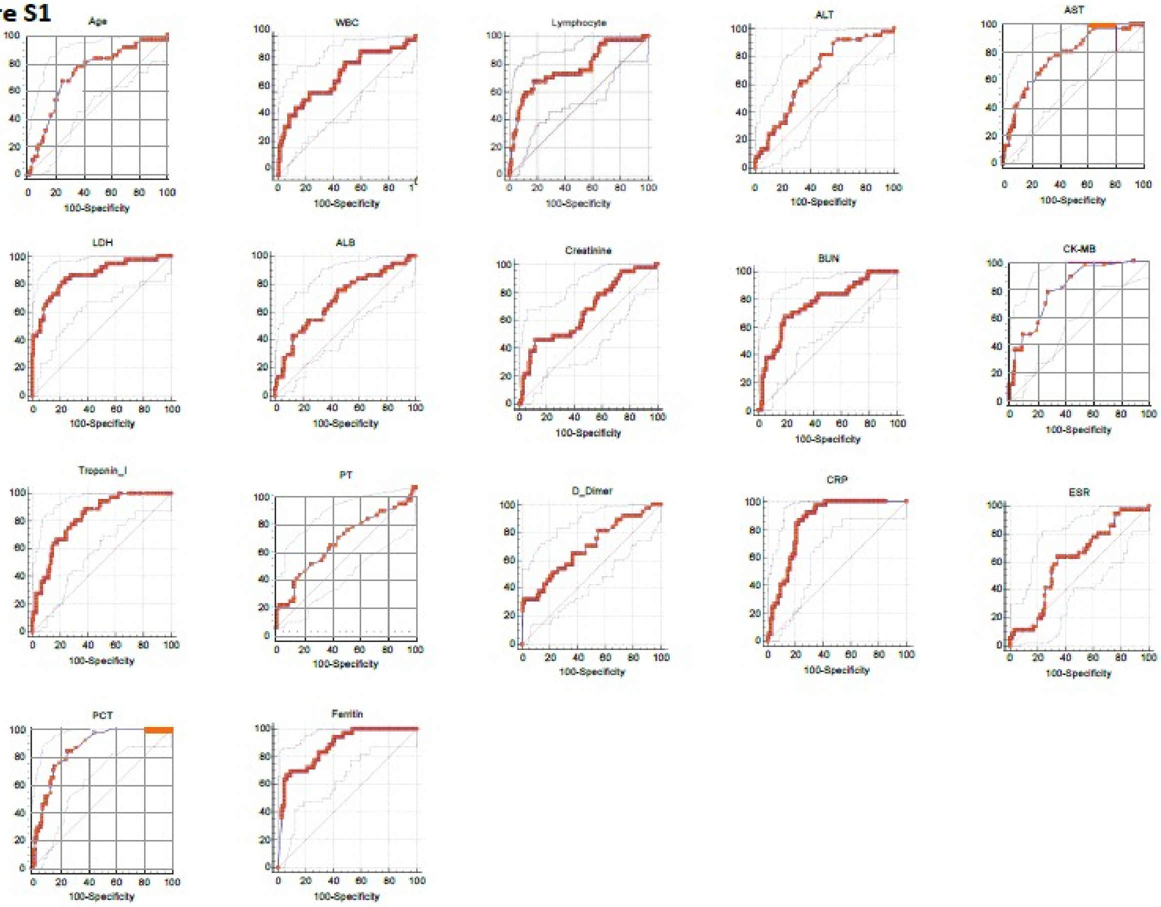
Age
WBC
Lymphocyte
ALT
AST
LDH
ALB
Creatinine
BUN
CK-MB
Troponin_I
PT
D_Dimer
CRP
ESR
PCT
Ferritin
100-Specificity
